# Characterization of Self-Compacting Concrete at the Age of 7 Years Using Industrial Computed Tomography

**DOI:** 10.3390/ma18194524

**Published:** 2025-09-29

**Authors:** Oana-Mihaela Banu, Sergiu-Mihai Alexa-Stratulat, Aliz-Eva Mathe, Giuseppe Brando, Ionut-Ovidiu Toma

**Affiliations:** 1 Faculty of Civil Engineering and Building Services, “Gheorghe Asachi” Technical University of Iasi, 700050 Iasi, Romania; oana-mihaela.banu@academic.tuiasi.ro; 2 Faculty of Civil Engineering, Technical University of Cluj-Napoca, 400641 Cluj-Napoca, Romania; aliz.mathe@mecon.utcluj.ro; 3 Department of Engineering and Geology, University “G. d’Annunzio” of Chieti-Pescara, 66100 Chieti, Italy; giuseppe.brando@unich.it

**Keywords:** internal pore structure, dynamic elastic properties, compressive strength, long age

## Abstract

The pore structure of SCC and of all cement-based materials plays a crucial role on the mechanical and durability characteristics of the material. The pore structure is affected by mix design, water–binder ratio and the incorporation of SCM and/or nanomaterials, all of which can improve mechanical and durability characteristics by decreasing porosity. Computed tomography (CT) is a powerful, non-destructive imaging technique to investigate the internal pore structure of concrete. The main advantage compared to other investigation techniques used to assess the pore structure is in terms of sample size. More specifically, industrial CT can be used to scan large concrete samples and be able to assess the internal pore structure without damaging the specimen. CT provides accurate measurements of pore diameters, volumes and shapes and enables the assessment of the total porosity. The paper presents the results of an experimental program on the characterization of self-compacting concrete (SCC) at a very long age (7 years) in terms of static and dynamic elastic properties and compressive and splitting tensile strength, all of which are correlated with the internal pore structure assessed via the use of an industrial Nikon XTH 450 CT. The results highlight the influence of pore volume, maximum pore diameter and sphericity on the strength and elastic properties of SCC at the age of 7 years. Both the compressive strength and the static modulus of elasticity values tend to decrease with the increase in the internal total porosity, with stronger influence on the former.

## 1. Introduction

Self-compacting concrete (SCC) is a new kind of concrete that can flow and settle on its own, filling formwork and covering reinforcement without the need for mechanical vibration. It possesses several advantages over the traditional, vibrated concrete, among which the following are worth mentioning: its ability to easily spread and fill the formworks with dense reinforcements, thus improving construction quality and speed [1]; it eliminates the need for mechanical vibrations reducing labor time and costs, noise pollution and the risk of segregation and/or voids due to improper vibration [2,3]; and its improved durability properties due to its dense microstructure [4,5] and reduced permeability [6].

In much the same way as with vibrated concrete, SCC can incorporate a wide variety of wastes to help reduce its carbon footprint and, at the same time, solve, at least in part, the problem of landfills. When fly ash and slag were used as supplementary cementitious materials (SCMs), a refined pore structure, improved mechanical properties and higher rates of strength gains were obtained [1,7,8]. The use of aeolian sand and recycled aggreges in precise dosages resulted in improved mechanical and durability characteristics of SCC [6]. Coal gangue powder and cement kiln dust were also successfully used as SCMs to obtain SCC with higher values of compressive strength and better microstructure [9].

The pore structure of SCC and of all cement-based materials plays a crucial role on the mechanical and durability characteristics of the material [10,11]. It has been proven that smaller, closely spaced pores are more efficient in relieving pressure due to ice formation during freezing [12], while larger and more distant pores may negatively affect the compressive strength [6]. SCC typically contains a large number of micropores, often in the range of 3–10 µm in diameter [13]. At the same time, it contains various pore types ranging from cylindrical, to ink-bottle shape, to planar interstitial pores [14]. There is a strong negative correlation between the porosity of SCC and its mechanical properties. Among the most significant pore characteristics affecting the properties of SCC are the specific surface area and the average chord length of the pores [15]. The pore structure is affected by mix design, water–binder ratio and the incorporation of SCMs and/or nanomaterials, all of which can improve mechanical and durability characteristics by decreasing porosity and detrimental pore types. Monitoring and optimizing pore structure is crucial for attaining high-performance, durable SCC for structural applications [9,16].

Mercury intrusion porosimetry (MIP) is one of the most popular techniques for determining the distribution of pore sizes. It is based on high-pressure injection of mercury into the sample, and this requires meticulous preparation. At lower pressure values, mercury fills the bigger pores but cannot fill the smaller ones. In order to fill inter- and intra-particle pores, high pressure is needed [17,18]. However, applying MIP to evaluate the pore structure of different media, including concrete, has several drawbacks. Significant differences were found between MIP and other assessment methods, such as helium pycnometry, as highlighted in one of the first studies to compare the two approaches [19]. It was concluded that mercury could not penetrate all the closed pores that helium would otherwise be able to enter. Moreover, subsequent research found that MIP might not be a perfect representation of the actual pore structure. The pore size distribution differed greatly from other approaches, even though the total porosity may have been accurately determined. When using MIP to evaluate the pore structure, several factors must be carefully considered, including the effect of ink bottle pores trapping mercury, the pore connectivity effect and the different contact angles between mercury and the solid portion of the samples during the intrusion and extrusion stages [20]. In addition to the very small material samples required to conduct the test, pore structure may potentially be harmed due to the very high pressure [21].

Low-field nuclear magnetic resonance (NMR) relaxometry is an advanced, non-invasive technique for characterizing the pore structure of porous media, including cement-based materials [22]. It is based on the differences in relaxation times of liquid molecules (typically water) within various pore environments, enabling detailed insights into pore size distribution and dynamics during material hydration [23,24]. Different pore sizes and types (gel pores, capillary pores, macropores) produce distinct relaxation signatures, allowing for the identification and quantification of multiple pore classes within the material. NMR has several advantages over MIP, such as the fact that it does not damage the sample and avoids the high pressures and toxic mercury used in MIP [25]; the samples can be analyzed in their native state, without the need for drying, cutting, or other preparation steps that might alter the pore structure [23]; it can be applied to fresh, hydrating cement pastes, enabling the study of pore development and water consumption as hydration progresses [22]; and the non-destructive nature allows for repeated measurements on the same sample over time. However, it also has some limitations that need to be acknowledged: precise assessment of absolute pore diameters necessitates the calibration of the pore surface relaxivity parameter, usually accomplished through comparison with MIP or scanning electron microscopy (SEM) data [26,27]; it is assumed that pores are fully saturated with water during measurements [23]; and similar to MIP, the tests are conducted on small scale samples, which makes generalizations rather difficult.

Computed tomography (CT) is a powerful, non-destructive imaging technique to investigate the internal pore structure of concrete [28,29] and aggregate size, shape and distribution, as well as fiber distribution [30,31]. The main advantage with respect to the above-mentioned methods is in terms of sample size. Unlike destructive techniques or laboratory-scale micro-CT, which are generally restricted to small specimens and may not represent the behavior of structural elements, industrial CT enables the scanning of large concrete specimens without damaging them. The distinction is paramount, as it allows pores to be characterized in samples closer to real casting conditions, bridging the gap between laboratory-scale determinations and the performance in a structural-scale situation. Industrial CT measurements provide the possibility to make quantitative assessments of pore diameter, volume, shape and total porosity. Given that the technique involves the 3D image analysis of a specimen that can be used in other tests (mechanical or otherwise), there is great opportunity to study the underlying impact of pore networks in durability and transport phenomena [32,33]. Moreover, total porosity has been deemed insufficient for explaining macroscopic properties, with pore morphology, size and distribution playing an important role [11], providing more incentive to further develop the full-scale CT analysis on concrete specimens. The 3D image analysis quantifies the complexity and interconnectivity of the pore networks, which are critical for understanding fluid transport and durability [29,34]. There are also limitations of the CT approach, which are mostly related to the scanning resolution and differentiating between pores and solid phases, which can be a challenge, especially for pores filled with water or other low-density materials [35]. It has been pointed out that the present technology is not a more important restriction than information interpretation and data processing. Even if traditional techniques are more apt, at the moment, in resolving pores smaller than air voids (under 10 µm), the possibilities offered by CT scanning might prove far greater [36].

Since the development of strength on concrete is strongly related to the pore structure, irrespective of the considered age, the present study brings its contribution to the assessment of strength and elastic properties of self-compacting concrete at a long age, 7 years, in close relationships to the pore structure. Previous studies showed that concrete mechanical properties improve over time and there is a tendency of pores to be filled by hydration products, thus reducing the number of large pores and the total porosity and modifying the pore shape [37,38,39]. The latter were assessed by means of industrial CT and the 3D reconstruction, which was conducted using the Dragonfly 3D World 2024.1 software. Although CT has been increasingly used in imaging cement-based materials, most existing studies have focused on small laboratory specimens or early-age concrete, most often relying on micro-CT or destructive testing [40,41,42]. The use of an industrial CT scanning equipment meant that entire concrete cylinders could be scanned [30], thus offering a broader image on the pore distribution inside the concrete specimens. This large-scale, non-destructive imaging approach allows a more representative evaluation of pore distribution, volume, diameter and sphericity, while preserving specimens for complementary mechanical testing. Three-dimensional reconstructions were performed using the Dragonfly software, enabling quantitative correlation between pore characteristics and static/dynamic elastic properties, compressive strength and splitting tensile strength.

## 2. Materials and Methods

### 2.1. Materials

A CEM II/A-LL 42.5R rapid hardening cement, produced by Heidelberg Cement (Bicaz, Romania), complying to currently available norms [43], was used in this research. It contains 80–94% clinker and 6–20% limestone filler, which prevents bleeding and ensures a proper hydration of the cement, and 0 ÷ 5% of other minor additional constituents. The chemical composition of the cement is presented in Table 1.

Limestone filler was also used as a mineral additive to increase the paste volume and avoid the use of viscosity modifying admixtures [44,45,46]. Natural river aggregates, from a local supplier, were used in the concrete mix as highlighted in [47]. The particle distributions for the aggregates are presented in [47].

The considered mix proportions, complying with guidelines for self-compacting concrete [48,49], are presented in Table 2. A Sika ViscoCrete—19 HE (Sika Romania, Bucharest, Romania) high range water reducer (HRWR), a polycarboxylate-ether based superplasticizer, was used as 0.5% by the cement mass. It has a specific gravity of 1.06 ± 0.02 g/cm^3^ and, according to the technical specifications, should be used in 0.3–2.5% by mass of the cement.

A constant 0.53 water/cement ratio was adopted for all 3 mixes, as seen in Table 1. The water-to-cement ratio of 0.53 was selected as a practical compromise to achieve the target self-compactability (slump-flow/passing ability) for the materials and superplasticizer dosages available in this study, while maintaining acceptable mechanical and permeability performance. Values of w/c between 0.4 and 0.55 were frequently reported in the scientific literature referring to self-compacting concrete [50,51]. Trial mixes were cast employing various combinations between w/c ratio and percentage of superplasticizer so that all 3 mixes will fall within the same slum flow class and V-funnel class [52].

### 2.2. Methods

A similar testing procedure reported in [53] was also adopted in this study. After casting, the 100 mm × 200 mm cylinders (diameter × height) were demolded 24 h later and cured in water for 28 days. Fourteen specimens for each mix proportion shown in Table 1 were further kept in laboratory conditions (23 ± 2 °C and 40–50% relative air humidity) until the day of testing, 7 years later.

The fresh properties of the SCC [47] were assessed by means of a slump flow test and T_500_ test [54], V-funnel test [55] and L-box test [56]. A higher slump flow diameter means the concrete spreads more freely, indicating better flowability. At the same time, a shorter T_500_ time signifies faster flow. A shorter flow time obtained from the V-funnel test suggests a more fluid and workable SCC mix that can easily flow and fill forms. The L-box test on the other hand assesses its passing ability, which is its capacity to flow through obstacles like reinforcement bars without blocking or segregating [57].

The open porosity of SCC mixes was obtained on 3 specimens from each mix presented in Table 1, following the guidelines of ASTM C642-21 [58]. The concrete cylinders were oven dried until constant mass (e.g., difference between two successive measurements would not differ by more than 0.5%). Afterwards, they were placed in water for no less than 48 h and their mass was measured again until constant mass in saturated conditions. The third step consisted in boiling the specimens for 5 h, cooling the specimens to room temperature while immersed in water, followed by their weighing. The volume of permeable pores (open porosity) was then computed.

The static modulus of elasticity was determined in accordance with SR EN 12390-13 [59]. In order to correctly set the stress limits mentioned in the code, 1 sample was loaded in compression until failure. Cyclic loading was applied to each of the remaining 9 specimens and three individual values were obtained for each concrete cylinder. The obtained values were compared to each other in order not to differ by more than 1% from one-another for the same concrete cylinder.

The longitudinal dynamic modulus of elasticity, the dynamic shear modulus and dynamic Poisson’s ratio were determined according to ASTM C215-19 [60]. The two dynamic moduli are based on the first resonant frequency (longitudinal and torsional frequencies, respectively) obtained from the Impact Echo Method. As presented in [53], the dynamic modulus of elasticity for the SCC, *E_d_*, was computed based on Equation (1):

(1) $$E_{d}=D\times m\times f_{ln}^{2}$$

where *m* is the mass of the cylinder, expressed in kg, *f_ln_* is the fundamental longitudinal frequency of vibration, expressed in Hz, and *D* is a shape coefficient that depends on the dimensions of the sample, as shown in Equation (2):

(2) $$D=5.093\times \frac{L}{d^{2}}$$

in which *L* is the length of the cylinder, in meters, and *d* is the diameter, given in meters.

On the other hand, the dynamic shear modulus, *G_d_*, was based on the fundamental torsional frequency of vibration, and was computed based on Equation (3):

(3) $$G_{d}=B\times m\times f_{t}^{2}$$

where *f_t_* is the fundamental torsional frequency of vibration, expressed in Hz, and *B* is a shape coefficient that depends on the geometrical characteristics of the sample, as shown in Equation (4):

(4) $$B=\frac{4LR}{A}$$

with *R* being a shape factor equal to 1 [60], and *A* is the cross-sectional area of the cylinder, expressed in m^2^.

The two dynamic moduli were then used to compute the dynamic Poisson’s ratio, *µ_d_*, by means of Equation (5):

(5) $$\mu_{d}=\frac{E_{d}}{2G_{d}}-1$$

The compressive and splitting tensile strength values were determined in accordance with SR EN 12390-3 [61] and SR EN 12390-6 [62], respectively. The compressive strength was determined of 5 samples whereas the splitting tensile strength was determined from the remaining 4 samples. The applied loading rates were 0.6 MPa/s (4.71 kN/s), for the determination of the compressive strength, and 0.05 MPa/s (1.7 kN/s), for the determination of the splitting tensile strength.

Two specimens of each mix were investigated through industrial computed tomography (CT) using a Nikon XTH 450 with 340 kV voltage and 240 µA current using a 3 mm copper filter. The detector had 2000 pixels × 2000 pixels and the pixel size was 40 µm, with a magnification factor of 2.27 and a white level of 60,000. The magnification factor represents the ratio of the image size to the actual object size or, equivalently, the ratio of the Source-to-Image-Distance (SID) to the Source-to-Object-Distance (SOD) [63]. The magnification factor is a phenomenon caused by the diverging nature of the X-ray beam, which spreads out from the source. The magnification factor was proven to significantly influence the quality of x-ray scans as compared to sampling resolution, which may result in higher computation effort and longer times to reconstruct scanned objects [64].

**Figure 1:**
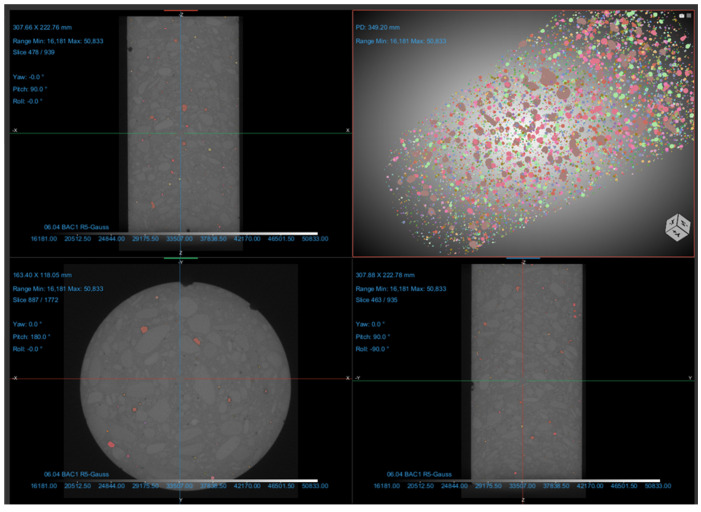
Determining internal pore structure and pore distribution in concrete cylinders.

The scanned samples were in dry conditions as pore water substantially impacts CT results by affecting image density, pore connectivity and the distribution of air–water phases within the scanned material. Its presence may induce imaging artefacts, complicating the precise characterization of pore structures [65]. Scan results were analyzed using the Dragonfly software, version 3D World 2024.1, which is specifically designed for 3D reconstruction of CT images, as well as for computing various parameters: porosity, sphericity of pores, volume and mean radius of pores and others. The procedure applied for the analysis of each specimen started with denoising the original CT result by applying a Gaussian filter [66]. Subsequently, the space surrounding the specimen was removed, as this is considered a pore by the software. This step results in also removing any surface or open pores (connected to the outside), so that only internal pores are left to be addressed [35]. An example of internal pore distribution within the specimen, along with different perspectives, is presented, in Figure 1.

The spacing in each direction (X, Y and Z) was 0.119591 mm for each of the specimens. The next step consisted in the segmentation procedure, in order to identify the pores. For this reason, the lower Otsu range was applied to the histogram. Given the resolution limitations, pores with a volume lower than 27 voxels (3 × 3 × 3 voxels) were eliminated. The focus was thus on pores that can be accurately distinguished, limiting the analysis to the air voids. These pores were further taken into consideration, as regions of interest (ROIs), for computing the amount that they represent out of the whole material volume [10,67]. Several parameters were extracted during the analysis: equivalent spherical diameter, mean pore radius, sphericity and volume.

## 3. Results

### 3.1. Fresh Properties of SCC

There is a strong, generally inverse correlation between slump flow and T_500_ time in self-consolidating concrete (SCC); as slump flow increases, T_500_ time (a measure of viscosity) typically decreases. A larger slump flow indicates better flowability (lower interparticle friction), while a longer T_500_ time signifies higher viscosity, meaning greater resistance to flow and a more cohesive mix. [9,68,69,70]. The fresh properties of the considered mixes are summarized in Table 3 [71].

It can be observed that all considered SCC mixes belong to the slump flow class SF2 and V-funnel class VF2. Moreover, they all comply to the European Guidelines for Self-Compacting Concrete [48], with a slump flow of 600–750 mm and T_500_ time of 3.5–6.0 s.

### 3.2. Bulk Density and Open Porosity

The bulk density was determined on all 14 specimens considered for each mix. The dimensions of the cylinders were measured using a digital caliper with an accuracy of 0.01 mm. Six values for the diameter and three values for the height were measured in order to compute the volume of each specimen. The mass was determined to an accuracy level of 0.01 g. The obtained results, as average of 14 values for each mix, are presented in Table 4.

At the same time, the open porosity was obtained as the average of three determinations, following the guidelines of ASTM C642-21 [58]. Similar values were reported in the scientific literature, although for earlier ages of SCC [16,72].

### 3.3. Static and Dynamic Elastic Properties

**Figure 2:**
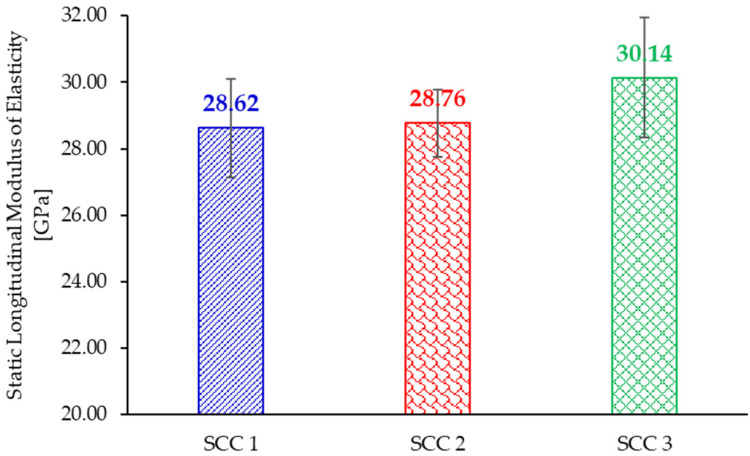
Static longitudinal modulus of elasticity for the considered SCC mixes.

The static and dynamic moduli of elasticity were determined on nine specimens for each SCC mix, as shown in Table 2. Figure 2 presents the static longitudinal modulus of elasticity in compression, Young’s modulus, for the SCC mixes. It can be observed that there is a gradual increase in the values of Young’s modulus with the increase in cement content.

**Figure 3:**
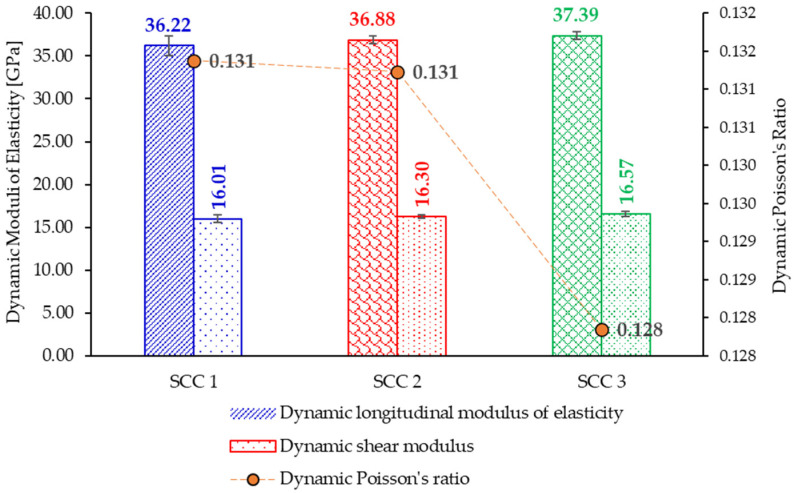
Dynamic elastic properties of the considered SCC mixes.

The dynamic moduli of elasticity, as well as the values of Poison’s ratio, are presented in Figure 3. The dynamic moduli of elasticity were determined based on the fundamental frequency of vibration, as presented in Table 5.

### 3.4. Compressive and Splitting Tensile Strength

**Figure 4:**
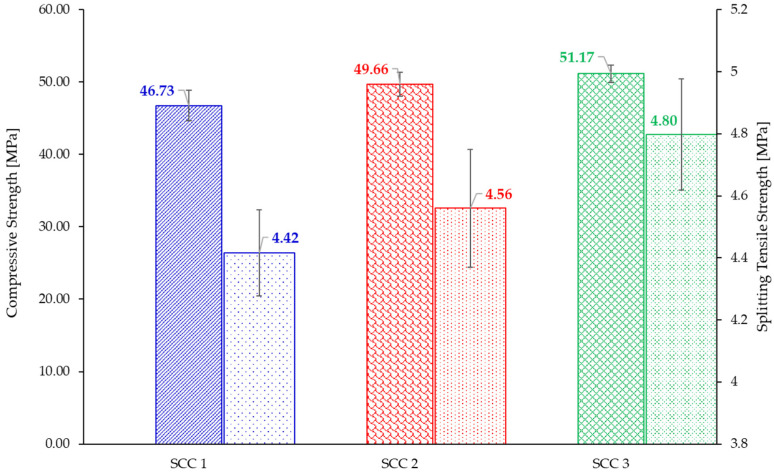
Mechanical properties of the considered SCC mixes at the age of 7 years.

The values of the compressive and splitting tensile strengths, determined at the age of 7 years, are presented in Figure 4. They represent the average of five and four determinations, respectively. The values of the mechanical properties follow the similar trend observed for the elastic properties, namely, the increase in the cement content resulted in an increase in the values of both compressive and splitting tensile strength.

However, the rate of increase in terms of compressive strength values is different than the increase in the splitting tensile strength values. An increase in the cement content and decrease in the aggregate mass, from SCC 1 to SCC 2, resulted in a 6.26% increase in the compressive strength and only a 3.26% increase in the splitting tensile strength. Further increasing the cement content while keeping the same aggregate mass and slightly decreasing the limestone filler, resulted in a further 3.06% increase in the values of the compressive strength and 5.22% increase in the values of the splitting tensile strength.

### 3.5. Internal Pore Structure

**Figure 5:**
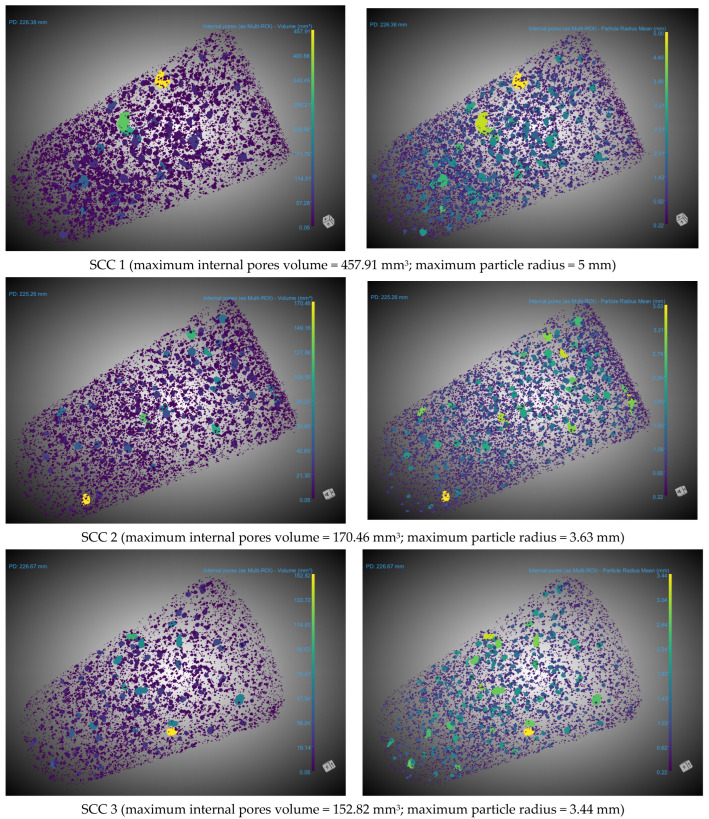
Internal pore structure of the considered SCC mixes at the age of 7 years.

The internal pore structure for each of the considered SCC mixes is shown in Figure 5. It can be observed that the total volume of the internal pores is decreasing with the increase in the cement content. A similar observation can be made for the particle radius value. The higher the cement content, the lower the particle radius due to the partial or total filling of the pores with hydration products [16,72]. Mechanisms that might explain this behavior, that are supported by experimental observations across several studies, relate to: (i) formation of fewer capillary pores; (ii) a denser structure of the hydration products; (iii) the filling of the voids between aggregates and paste; and (iv) a denser, less porous cement matrix [40,73].

## 4. Discussion

The changes in the mix proportions did not result in large variations of SCC density at the age of 7 years (less than 1%). This was also proven by the similar open porosity percentages of each considered SCC mix. Considering the long age, any differences that could have existed were gradually reduced due to the continuous hydration of the cement particles, thus creating additional hydration products [38,69].

### 4.1. Static and Dynamic Elastic Moduli

The evaluation of the dynamic modulus of elasticity offers the benefit of a non-destructive approach and can typically be conducted on-site. For design and technical evaluation, the static modulus of elasticity values were utilized, prompting the proposal of conversion equations. Previous studies [53,74] highlighted the suitability of several conversion formulas from the dynamic modulus of elasticity to the static one [75,76,77].

**Figure 6:**
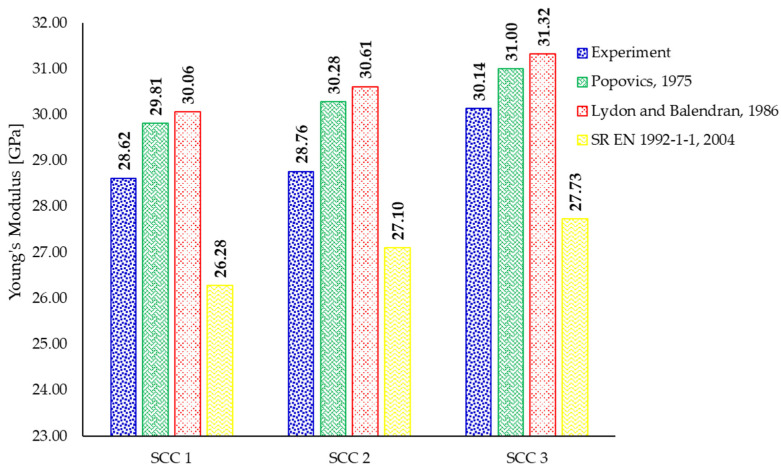
Prediction of the static modulus of elasticity by currently available equations [75,76,77].

The obtained results are summarized in Figure 6. It can be observed that both equations proposed by Popovics [75] and Lydon and Balendran [76] slightly overestimate the experimental results by 2.85–5.28% and 3.92–6.42%, respectively. On the other hand, the conversion equation currently available in the design code [77] tends to underestimate the experimental data by 5.79–8.18%.

### 4.2. Compressive Strength

**Figure 7:**
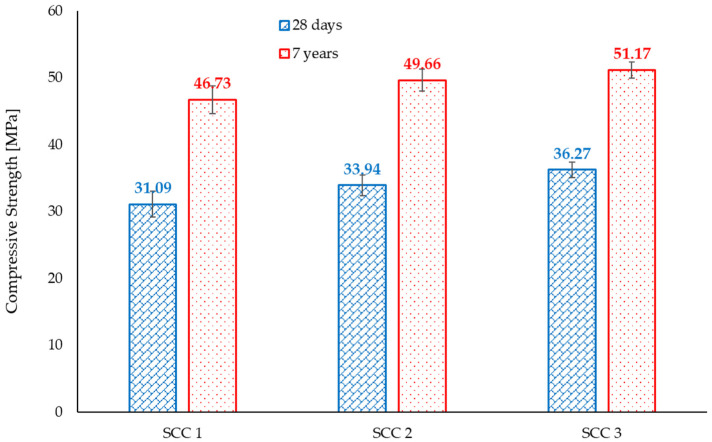
Evolution of the compressive strength.

The evolution of the compressive strength from the standard age of 28 days [47] up to 7 years is presented in Figure 7. As generally expected, the strength characteristics of concrete tend to improve over time due to continuous hydration of the cement particles. However, strength evolution is highly dependent on the storage/environmental conditions.

It can be observed that although the values of the compressive strength increased for all three SCC mixes, the rate of increase was not similar, ranging from a 50.3% increase for SCC1 to a 41.09% increase for SCC 3. Limestone filler interacts with the hydration products of cement, mainly via a filler effect and nucleation, as well as through chemical interactions [78,79]. These interactions can influence the initial strength development, microstructure, and long-term durability of the concrete [80]. However, at higher limestone contents, the dilution effect may become more prominent and may influence the long-term strength gains of concrete.

### 4.3. Pore Structure

Current CT technology predominantly emphasizes the precise identification of internal pores or cracks within concrete. Nonetheless, it is crucial to acknowledge that exterior pores and cracks on the surface exposed to the environment can considerably influence material strength. Although there is a significant number of research works addressing the porosity of cement based materials [11], it is still challenging to characterize such materials solely based on the total porosity [81,82,83].

One other significant parameter is the sphericity of the pores, that is, how close the pores are to the shape of a perfect sphere [84]. This perfect spherical shape of the pores helps reducing the stress concentrations under loading conditions. From the results obtained via the use of the Dragonfly software, it resulted that for each of the three considered SCC mixes, more than 95% of the identified pores had a sphericity of 0.99, that is they are very close in shape to a perfect sphere (sphericity = 1). A sphericity close to one for all mixes suggests that the detected pores are almost perfect spheres. This is to be expected in self-compacting concrete, given its very good fluidity, use of a superplasticizer and minimal external deformation during casting. These observations are consistent with recent studies showing that surface tension dominates deformation of air voids [85,86].

**Figure 8:**
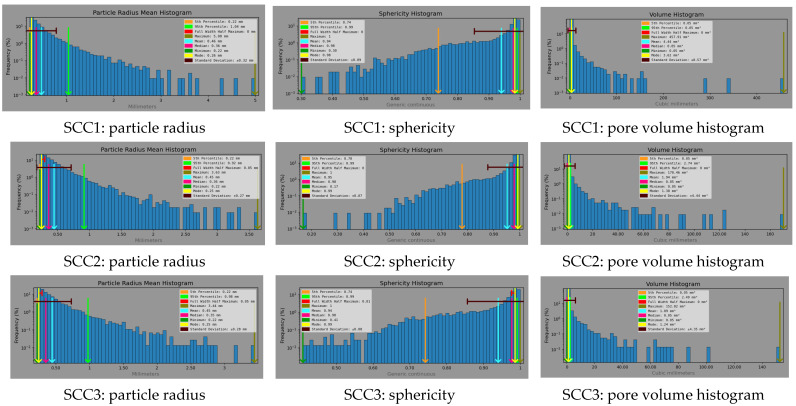
Internal pore structure characterization.

Figure 8 presents the distribution of particle radius, sphericity and volume of pores for all considered SCC mixes. It can be observed that increasing the cement content results in a denser matrix [11], with a decrease in the pore radius of the considered mix. While the large majority of detected pores in the SCC1 mix had a radius of up to 3 mm, for both the SCC2 and SCC3 mixes it was reduced to 2.5 mm and 2.00 mm, respectively [87].

At the same time, it can be seen that the values of pore sphericity increase with the increase in cement content.

At the same time, considering the data presented in Figure 5, it can be concluded that with the increase in the cement content of SCC, which leads to an increase in the values of the mechanical properties, the volume, as well as the maximum pore diameter, of the pores at the age of 7 years decreases. This could be explained by the fact that more hydration products are generated due to both higher cement content and due to the nucleation site effect of limestone filler.

Total internal porosity (φ) was computed as the void volume fraction relative to the full volume. Two classical porosity–strength models were evaluated and fitted by linear regression on logarithmically transformed variables. The results are summarized in Table 6 for compressive strength, static modulus of elasticity and dynamic modulus of elasticity.

**Figure 9:**
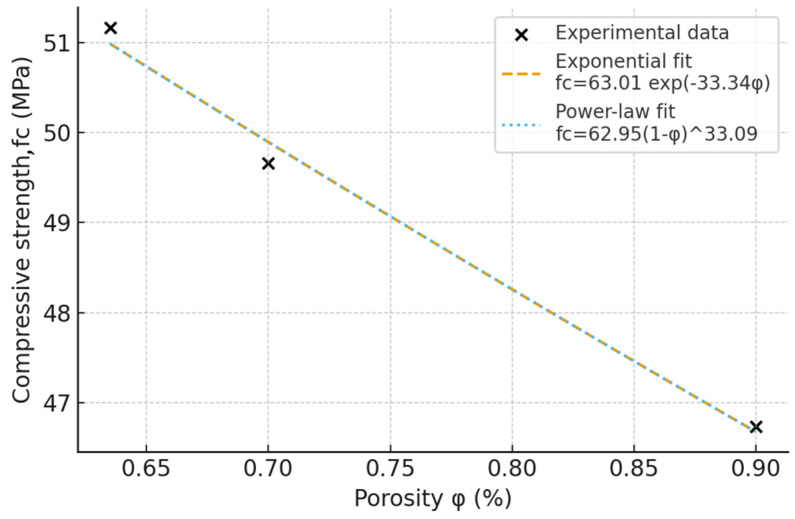
Influence of total internal porosity on the compressive strength.

**Figure 10:**
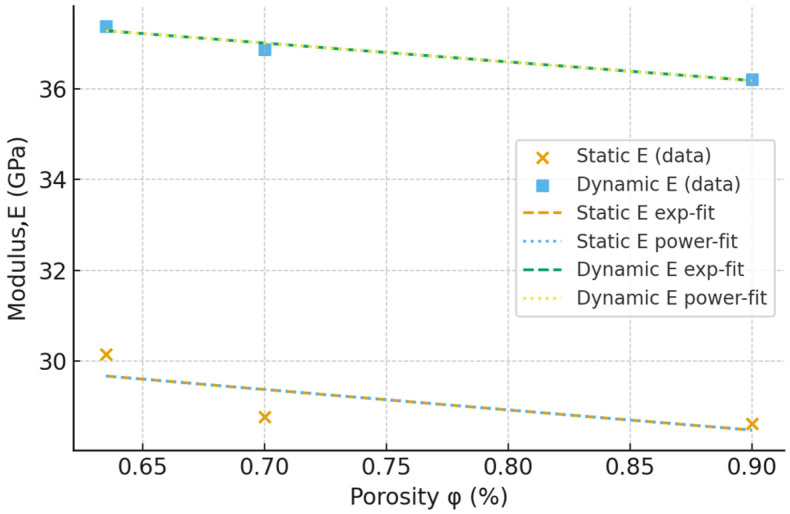
Influence of total internal porosity on the static modulus of elasticity.

The change in the elastic and mechanical property values of the SCC as a function of total internal porosity is presented in Figure 9 and Figure 10 for the compressive strength and static modulus of elasticity, respectively. However, given the small number of data sets, the presented results should be regarded as representing the trends. Further investigations are deemed necessary to correctly assess the influence of the internal porosity on the mechanical characteristics of the SCC.

## 5. Conclusions

The paper presents the results obtained on the characterization of self-compacting concrete at a long age (7 years) by using industrial computed tomography to assess the internal pore structure. Based on the obtained results, the following conclusions can be drawn.

1. There is little difference between the bulk density values of the considered SCC mixes at the long age. At the same time, the open porosity ranges from 8.54% to 12.9% and it is influenced by the cement content of the mix.
2. Compressive strength values increase compared to the standard testing age of 28 days. The increase rate is, however, inversely proportional to the cement content.
3. There is a good correlation between the conversion equations from the dynamic modulus of elasticity to the static one. Some of the existing equations tend to slightly overestimate the experimental results, while others underestimate the results.
4. The increased cement content and lower aggregate volume results in a lower volume of internal pores, as well as lower maximum pore radius, as determined through industrial CT scanning on the intact SCC cylinders. At the same time, the sphericity of the internal pores does not seem to be significantly influenced by the SCC mix proportion. Increasing the cement content above 340 kg/m^3^ has little influence on the internal pore structure of the SCC.
5. The total internal porosity has a more significant influence on the compressive strength of SCC mixes than on the static modulus of elasticity. The values of the latter decrease at a much lower rate with respect to increasing internal porosity compared to the compressive strength.

## Figures and Tables

**Table 1:** Chemical composition of CEM II/A-LL 42.5R (expressed in %).

CaO	SiO_2_	Al_2_O_3_	Fe_2_O_3_	MgO	SO_3_	Na_2_O	K_2_O	MgO	TiO_2_
61.54	18.92	4.86	2.5	1.9	3.1	0.25	0.98	1.89	0.17

**Table 2:** Mix proportions for self-compacting concrete [47].

Concrete Mix	Cement	Sand [0–4 mm]	Aggregates [4–16 mm]	Limestone Filler	Water/Cement	Water/Powder *	HRWR
	[kg/m^3^]	[kg/m^3^]	[kg/m^3^]	[kg/m^3^]	-	-	[L/m^3^]
SCC 1	320	814	881	160	0.53	0.35	4.5
SCC 2	340	809	876	150		0.38	5.1
SCC 3	360	809	876	130		0.34	5.1

* cement + limestone filler.

**Table 3:** Fresh properties of the SCC mixes.

Concrete Mix	Sump Flow	T_500_	V-Funnel	L-Box
	[mm]	[s]	[s]	
SCC 1	680	4.2	14.4	0.84
SCC 2	690	4.1	11.3	0.88
SCC 3	720	2.9	9.2	0.93

**Table 4:** Bulk density and open porosity values.

Concrete Mix	Bulk Density	StDev	COV	Open Porosity
	[kg/m^3^]	[kg/m^3^]	[%]	[%]
SCC 1	2278	11.05	0.49	12.9
SCC 2	2291	7.10	0.31	11.08
SCC 3	2300	11.99	0.52	8.54

**Table 5:** Fundamental frequency of vibration (longitudinal and torsional) [60].

Concrete Mix	Longitudinal Frequency of Vibration	StDev	COV	Torsional Frequency of Vibration	StDev	COV
	[Hz]	[Hz]	[%]	[Hz]	[Hz]	[%]
SCC 1	9954.9	142.98	1.44	6628	79.04	1.19
SCC 2	10,030.3	53.73	0.54	6668.4	45.41	0.68
SCC 3	10,082	99.12	0.98	6712.4	40.26	0.6

**Table 6:** Properties of the SCC versus internal porosity.

Concrete Mix	Internal Porosity	Compressive Strength	StDev	COV	Static Modulus of Elasticity	StDev	COV
	[%]	[MPa]	{MPa]	[%]	[GPa]	[GPa]	[%]
SCC 1	0.9	46.73	2.11	4.51	28.62	1.47	5.14
SCC 2	0.7	49.66	1.65	3.33	28.76	1.02	3.54
SCC 3	0.635	51.17	1.19	2.33	30.14	1.81	6.01

## Data Availability

The raw data supporting the conclusions of this article will be made available by the authors on request.
